# Suicide Risk Management in Ecological Momentary Assessment: Participant Concealment of Suicidal Thoughts and Experiences with Safety Procedures

**DOI:** 10.1016/j.jad.2025.120361

**Published:** 2025-10-07

**Authors:** Anne C. Knorr, Brooke A. Ammerman

**Affiliations:** a University of Notre Dame, Department of Psychology, Notre Dame, IN, USA; b University of Wisconsin, Madison, Department of Psychology, Madison, WI, USA

**Keywords:** Underreport, Suicidal ideation, Experience sampling, Risk protocols, Research ethics, Participant safety, EMA

## Abstract

Ecological momentary assessment (EMA) has advanced the study of real-time suicide risk; however, the ethical obligations of researchers in responding to potential participant safety concerns are a topic of continued discussion. Initial research found that participants may modify their EMA ratings to avoid researcher contact, but the rates of underreporting or concealing of suicidal ideation (SI) are unknown, as are motivations for and correlates of these thoughts and behaviors. Beyond impacting participant reports, suicide risk assessment in response to elevated participant risk may also introduce intervention effects into EMA studies. This line of research is imperative to understanding barriers to ecologically valid data while also protecting participants. This study sought to fill these research gaps among a sample of community participants reporting past 6-month SI (*N* = 94; *M*_age_ = 33.63; 62.80 % female, 78.70 % white) who completed an EMA period and follow-up measures as part of a larger study. Underreporting and concealing SI was common during EMA. Multiple concerns motivated underreporting/concealing and several characteristics influenced the likelihood of this. Smaller within-person increases in SI were experienced when a risk assessment was completed, versus not, and a higher-than-usual SI intensity was reported prior to the assessment (Cohen’s *d* = 0.39). Survey responses following heightened or average within-person positive affect reflected smaller increases in reported positive affect when an assessment was completed, versus not (Cohen’s *d* = 0.49, 0.33, respectively). Although, many participants retrospectively believed the impact was more considerable and prolonged. Finally, participants provided multiple suggestions to mitigate the underreporting and concealment of SI.

## 1. Introduction

Suicidal ideation (SI), thoughts of killing oneself or wanting to be dead, is experienced by more than 12 million adults in the United States (Liu et al., 2020; SAMSHA, 2022) and is a pre-cursor to and predictor of death by suicide (Franklin et al., 2017; Liu et al., 2020). Ecological momentary assessment (EMA) is a promising avenue for improving our understanding of real-time SI (Ammerman and Law, 2022; Kivelä et al., 2022; Sedano-Capdevila et al., 2021). Through the repeated collection of fine-grained data measuring recent experiences without researcher involvement, EMA supports ecological validity and minimizes retrospective recall bias (Shiffman et al., 2008). Yet, ethical concerns accompany EMA application within suicide research, prompting the implementation of safety protocols sensitive to identifying increases in participant suicide risk (Bentley et al., 2021; Hoelscher et al., 2025; Kleiman et al., 2019; Nock et al., 2021; Schatten et al., 2020). These protocols, which vary (Bentley et al., 2021; e.g., ranging from resource reminders to risk assessment/safety planning), are intended to protect participants and prevent crisis escalation. Yet, concerns about participant behavioral reactivity to these protocols exist (Bentley et al., 2021), emphasizing the importance of exploring the extent to which safety protocols may impact the ecological validity of EMA data within non-interventional, suicide research.

First, the influence of EMA safety protocols on participants’ SI concealment – denying SI despite its presence – and underreporting – under-rating the intensity of present SI – remains poorly understood (Bentley et al., 2021; Nock et al., 2021) and no study has systematically studied the motivations behind and contributors to these thoughts and behaviors. Advances in understanding SI prediction, course, and characteristics (Kivelä et al., 2022; Kleiman et al., 2017; Kleiman et al., 2018) and the potential for clinical translation depend on the quality of collected data. The underreporting and concealment of SI within EMA research seeking to study such thoughts can compromise the ecological validity of the data and potentially lead to biased conclusions about the predictors, course, and variability of SI. Given that EMA is often used to identify near-term risk factors, inaccurate reporting could distort the temporal associations between risk markers and SI, misinform the development of predictive models, and limit translational potential. Likewise, a research team’s ability to detect high-risk states and engage in safety protocols relies on SI disclosures. Some individuals, including prior to suicide attempt, conceal SI when interacting with mental health and healthcare professionals (Blanchard and Farber, 2016; Love and Morgan, 2021). Only one study, to our knowledge, evaluated participant SI reporting during EMA (Bentley et al., 2024), accentuating the need for future research. This study found no change in survey completion following researcher intervention for high suicidal intent; yet, participants commonly underreported suicidal intent to stay below a researcher intervention threshold. Thus, participants may conceal or underreport SI to evade EMA safety protocols. However, rates of this are unknown. Additionally, non-EMA research suggests that common motivations for concealing SI are fear of overreaction and negative outcomes (e.g., psychiatric hospitalization; Blanchard and Farber, 2018; Hom et al., 2017; Richards et al., 2019); it remains unclear whether these concerns operate similarly within EMA studies. The identification of participant experiences (e.g., prior hospitalizations) and characteristics (e.g., demographics) associated with thoughts of and engagement in underreporting and concealing SI during EMA would further inform strategies to reduce barriers to accurate reporting.

Second, the implementation of EMA safety procedures may impact participant thoughts and emotions, yet the extent is unknown. EMA safety protocols often include researcher-conducted suicide risk assessments and/or safety planning (Bentley et al., 2024; Schatten et al., 2020), which may introduce treatment effects (Blades et al., 2018; Stanley et al., 2015; Stanley et al., 2018). Therefore, it is important to evaluate within-person changes in near-term SI and affective experiences following a suicide risk check-in. While most research has found minimal evidence of harm following repeated SI assessment (e.g., Blades et al., 2018), the possibility that suicide risk check-ins alter participants’ affective or cognitive states raises important questions about unintended intervention effects, even in observational contexts. These effects, although subtle, may confound efforts to understand the naturalistic dynamics of suicide risk. This research is needed to improve understanding of the potential residual, therapeutic effects of safety procedures and the subsequent influence on ecological validity.

Third, research is needed to explore participant viewpoints that may inform the development of study procedures to increase comfort with reporting SI during EMA. Recently, experts have published guidance for designing research to study suicide risk, including EMA (Nock et al., 2021; Schatten et al., 2020). Yet, to our knowledge no study has incorporated participant perspectives into recommendations. Obtaining participant input is a critical next step to empower participants and guide researchers in designing studies that may better preserve ecological validity while protecting participants.

To address these gaps, the current study examined participant experiences during an EMA study that monitored suicide risk and employed a safety protocol. First, this study aimed to quantify and contextualize concealment and underreporting of SI during EMA, including motivations and correlates. Second, this study evaluated changes in SI and emotion states following researcher-initiated suicide risk check-ins. Third, this study gathered participant recommendations for enhancing comfort with SI reporting during EMA. Given the exploratory nature of this study, there were no a priori hypotheses. These insights have the potential to inform future study designs that protect participants while preserving ecological validity, thereby improving data quality and suicide risk detection.

## 2. Method

### 2.1. Participants and procedures

**Fig. 1.**
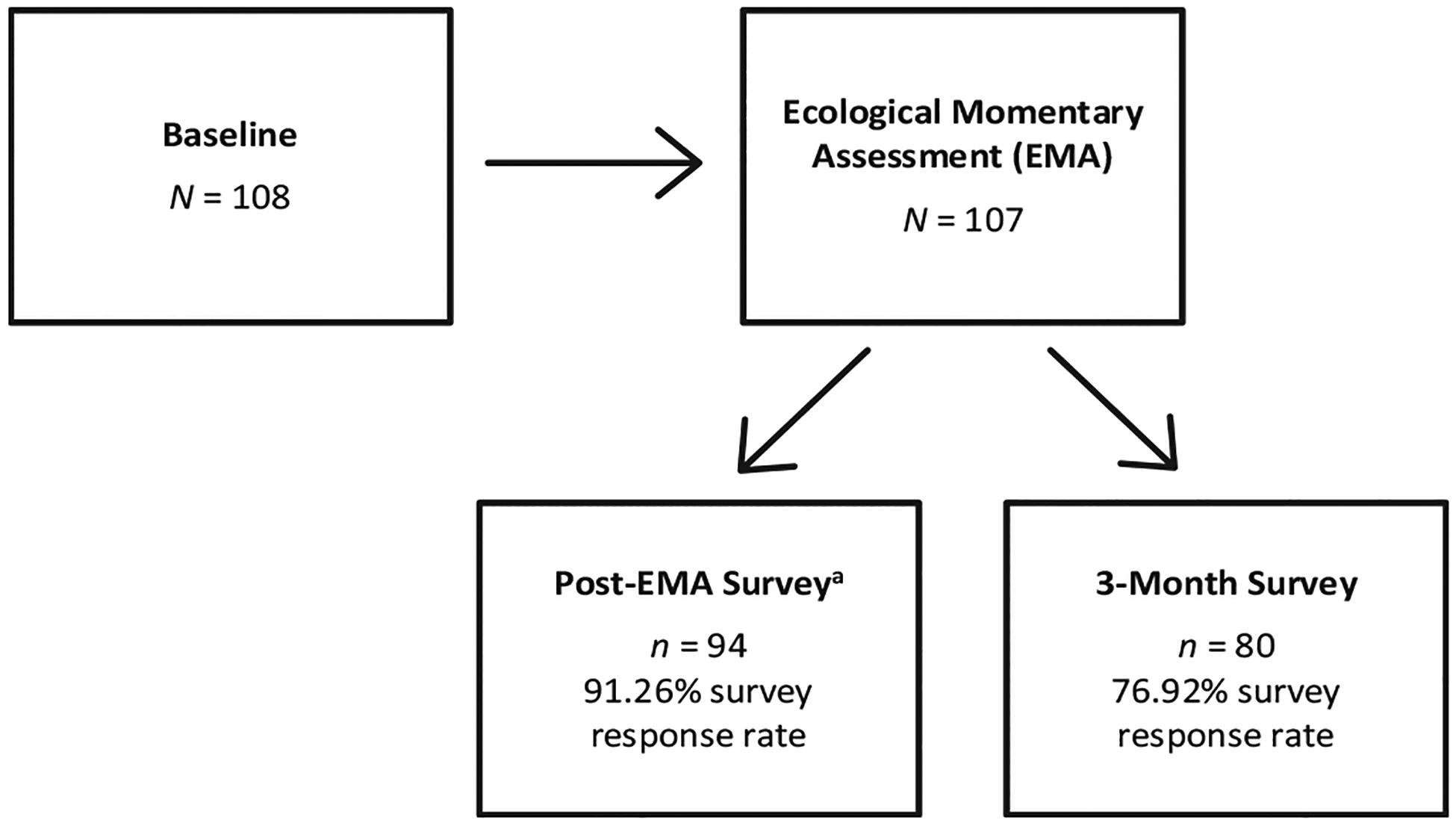
Study participation, retention, and response rate *Note*. ^a^75 (79.79 %) participants completed both the post-EMA and 3-month surveys.

Participants with any past 6-month history of active SI (i.e., thoughts of killing oneself) with at least 2 total lifetime experiences of SI were recruited using social media and community advertisements for participation in a larger study (*N* = 108 consented). Participants completed a 1.5-h virtual baseline session and a 30-day EMA period, with the option of a post-EMA survey assessing experiences about researcher check-ins to evaluate suicide risk (*n* = 103 provided consented to receive this survey) and a 3-month survey asking for suggestions regarding future EMA work (*n* = 104 consented). See Fig. 1 for a summary of participation in each study phase, including retention and response rate details. All available data from individuals who completed the post-EMA (*n* = 94) or 3-month (*n* = 80) surveys was used. Sample characteristics for those who completed the post-EMA survey are presented (Table 1), given the overlap of participation in this and the 3-month survey. Age ranged from 18 to 72 years; participants were predominantly female, non-heterosexual, employed, and white (78.70 %; black, 7.40 %; asian, 3.20 %; american indian/alaskan native, 2.10 %; multiple, 5.30 %; other, 3.20 %). Participants were compensated up to $170. This study was approved by the University of Notre Dame (protocol #23-08-8039).

During informed consent a detailed script (see Supplemental Material 1) was utilized to ensure consistency of provided information regarding the limits of confidentiality related to suicide risk, research team clinical expertise, and description of the safety protocol (e.g., if a participant could not be reached by phone for a check-in, there is no further action). During the baseline session, participants received a mental health resource list and active SI recency and frequency was assessed using the Self-Injurious Thoughts and Behaviors Interview (Nock et al., 2007) to evaluate study eligibility.

During the EMA period participants received survey notifications on their smartphones three times per day (~3–4 min/survey), randomized within blocks across 12 h. Participants reporting any active or passive SI (responses ≥2 out of 5) received an in-app reminder of crisis resources. Reports of active SI at a moderate-to-high intensity (responses ≥3 out of 5) triggered a phone contact attempt by the research team within 12 h (i. e., trained graduate student or faculty investigator, who is a licensed psychologist), with three contact efforts made to complete a suicide risk check-in and resources emailed. Phone calls were the primary check-in method, with text messaging used if a participant expressed a preference.

If direct contact was made, the participant’s current physical location during the present interaction was obtained in case of emergency, with participants informed that this information would be deleted at the end of each interaction and would not be kept on file. A comprehensive suicide risk assessment was then conducted using risk stratification from established clinical risk models (U.S. Department of Veteran Affairs, 2024). Although clinical judgement was used during the check-in, general decision-making was guided by pre-set criteria. If participants were determined to be at low acute risk (i.e., no present SI, suicidal intent, or planning reported, high confidence to maintain safety), resources were reviewed. If intermediate acute risk was determined (i.e., SI reported without plan and ability to maintain safety), a safety plan was developed or reviewed (Stanley et al., 2018) and emailed following contact. If high acute risk was determined (i.e., high intent to die by suicide and inability to maintain safety), a collaborative plan was created for the participant to seek in-person suicide risk assessment (emergency department transport/calling 911); if a participant was unwilling to follow this recommendation or the call was disconnected and the participant unreachable, the protocol specified that the researcher should request a safety evaluation from emergency services to the address provided by the participant during the contact. If a participant did not complete a researcher check-in when contacted, no additional steps were taken given lack of evidence of imminent suicide risk from study data (i.e., surveys only assessed SI). All participant contact related to the risk management protocol was documented.

### 2.2. Measures

#### 2.2.1. Baseline

Participants reported demographic characteristics and treatment history (past-year outpatient psychiatric treatment, yes/no; number of psychiatric hospitalizations). Participants also completed the Self-Injurious Thoughts and Behaviors Interview (Nock et al., 2007); age of active SI onset, active SI lifetime frequency, suicide plan history (yes/no), and suicide attempt history (yes/no) were utilized in the present analysis.

#### 2.2.2. EMA

Four items assessed momentary (‘At this moment’) passive (“Life is not worth living”; “There are more reasons to die than to live for me”) and active SI (“I want to die”, “I think about taking my life”), with ratings from 1 (*not at all*) to 5 (*very much*; Forkmann et al., 2018); responses to the four items were summed as a measure of SI intensity (i.e., score from 4 to 20). The percentage of EMA surveys with any SI reported, defined as rating any active or passive SI item as two or greater on a survey (i.e., any non-zero SI), was calculated to measure the frequency of reporting SI during EMA. No other aspects of suicidality were measured. Six items (1 = *not at all*; 5 = *very much*) assessed positive and negative momentary affective states (Forkmann et al., 2018), with sum scores computed for positive affect (“cheerful”, “happy”; i.e., score from 2 to 10) and negative affect (“afraid”, “nervous”, “downhearted”, “sad”; i.e., score from 4 to 20). Participant EMA compliance (percentage of completed surveys) was also measured.

#### 2.2.3. Safety protocol tracking

Implemented safety procedures and outcomes were recorded and used in the present study, including: (1) attempted contact to complete a suicide risk check-in (yes/no), (2) type of contact used (call/text), (3) contact date/time, (4) contact outcome (suicide risk assessment check-in completed; responded to contact but check-in was not completed due to unavailability or lack of willingness; no response), and (5) additional intervention(s) utilized (e.g., safety plan creation/review, arranged emergency department transport). This data was used to calculate the number of completed suicide risk assessment check-ins per participant (either by call or text) and to categorize participants based upon level of contact with the researcher over the course of the study (mutually exclusive): completed ≥2 check-ins; completed 1 check-in; responded to researcher contact but never completed a check-in; did not respond to researcher contact (i.e., never replied).

#### 2.2.4. Post-EMA survey

Please see Supplemental Material 2 for a copy of the post-EMA survey. Thoughts of and engagement in underreporting and concealing SI during EMA was measured. Participants were asked if they recalled experiencing “suicidal thoughts” while completing an EMA survey. Those who recalled this were subsequently asked if at some point they thought about underreporting SI (i.e., “considered reporting a lower number than was accurate”; yes/no) and concealing SI (yes/no) on an EMA survey, as well as how often they engaged in these behaviors (never, once, more than once). Based upon these responses, two additional variables were created to measure having thoughts of (yes/no) and engaging in behaviors of (yes/no) modified reporting (i.e., thoughts or behaviors of underreporting *and/or* concealing, respectively) of SI during EMA. Additionally, those who reported any prior *thoughts* of underreporting or concealing SI were asked to endorse all applicable reasons motivating these thoughts (i.e., did not want to be contacted, fear of police involvement, fear of involuntary hospitalization, ‘other’).

Participants were also asked to report on the typical influence of completed researcher check-ins on decreasing “suicidal thinking”, decreasing “negative thoughts or emotions”, and increasing “positive thoughts and emotions” (all rated from *not at all* to *extremely*, with the option to indicate absent SI at contact). The duration of these experiences was also reported (i.e., from ‘during the check-in’ to ‘more than 2 days after’). To better understand engagement in other interventions, participants were asked to report their use of (yes/no) mental health crisis resources during the study and aspects that influenced this (e.g., receiving resources from app/researchers, completing check-in, etc.).

#### 2.2.5. 3-month survey

One free-response item from the 3-month survey was used in the current study: “What, if anything, would have made you feel comfortable to report current suicidal thoughts on the daily surveys? Detailed responses will help us decide the best way to design our future studies”.

### 2.3. Data analysis

To increase statistical power, measures of race, sexual orientation, and employment status were dichotomized due to small cell sizes. Multilevel modeling, as described below, was conducted in the R statistical environment 4.4.2 (R Core Team, 2024; specific packages noted below), with IBM SPSS version 29 (IBM Corp., Armonk, N.Y., USA) used for all other analyses.

#### 2.3.1. Aim 1: Evaluate concealing and underreporting suicidal ideation during EMA

When analyzing data from the post-EMA survey concerning SI concealment and underreporting, there were 11 (11.96 %) participants with missing data due to having no recollection of reporting SI (passive or active) on at least one EMA survey. Thus, data was reported for 81 participants, with two choosing not to answer questions related to contemplating and engaging in SI concealment. A series of chi-square, Fisher’s exact (when appropriate), independent samples *t*-tests, and Welch’s t-test (if equal variances assumption was violated) analyses were conducted to investigate correlates (see Table 1 for variable list) of having thoughts of modifying SI reporting and also engaging in modified SI reporting. Effect sizes are reported and interpreted based upon established standards (Cohen, 1988; Kim, 2017), including Cohen’s *d* for pairwise comparisons (small = 0.2, medium = 0.5, large = 0.8) and Cramer’s *V* for chi-square tests (e.g., with 1 degree of freedom: small = 0.1, medium = 0.3, large = 0.5). Multivariable models were not constructed due to concerns related to power and overfitting. This study’s sample size does not meet the minimum number of participants required for between-person logistic regression (*n* = 15 predictors), as per existing standards (Austin and Steyerberg, 2017; Bujang et al., 2018; Peduzzi et al., 1996; Steyerberg et al., 2000).

#### 2.3.2. Aim 2: Investigate the influence of the EMA safety protocol on momentary suicidal thoughts and emotions

Safety protocol tracking data was utilized to identify the start date and time of a telephone call or text check-in that was completed to evaluate participant suicide risk. The date and time were cross-referenced with the EMA data. The next EMA survey following the start date/time of the check-in was identified as *time t* + *1* (i.e., survey after the suicide risk check-in), with the EMA survey prior coded as *time t* (i.e., survey before the check-in). Those who had not completed a check-in following a survey were coded as “no” for the check-in variable. Then, changes in participant momentary SI (6425 surveys, *N* = *9*1), negative affect (6650 surveys, *N* = 92), and positive affect (6669 surveys, *N* = 92) before (i.e., *t*) and after (i.e., *t* + *1*) completing a suicide risk check-in were evaluated using three separate multilevel models – one per outcome – with repeated observations (level-1) nested within-person (level-2). For example, the global SI score at *time t*, the variable measuring check-in completion (i.e., between *time t* and *t* + *1*; 1 = yes, 0 = no), and the interaction of SI x check-in were included as focal predictors, with survey number at *time t* included as a time-varying covariate (to control for time effects), and global SI score at *t* + *1* (i.e., time point after the check-in) included as the outcome. This same structure was repeated for the multilevel models focused on negative affect and positive affect (i.e., in each model the same construct was included as the predictor *and* outcome, with affect x check-in included as a predictor). SI, negative affect, and positive affect were each included as fixed and random effects in their respective models. The primary predictors (SI, negative affect, positive affect) were each person-mean centered (Wang and Maxwell, 2015).

The *robustlmm* package in R (Koller, 2016) was used to analyze the SI model using robust standard errors due to deviations from normality for the outcome (skew = 2.5, kurtosis = 8.28), with the *confitROB* package also used (to generate 95 % confidence intervals [CI]; Mason et al., 2024). The *Lme4* package (Bates et al., 2015) was used to analyze the negative and positive affect models, in conjunction with the *stats* package (95 % CIs; R Core Team, 2024). The *emmeans* (Lenth, 2025) package was used to explore significant interactions; at each level of the moderator (check-in, yes/no), the estimated marginal means of the outcome were compared at different levels of the predictor (one standard deviation below the mean [lower-than-usual], the mean [average levels], and one standard deviation above the mean [higher-than-usual]). The influence of *attempted* check-in contact on SI and affect was not investigated as the date/time at which a participant became aware of a contact attempt is unknown.

#### 2.3.3. Aim 3: Explore participant recommendations to increase comfort with reporting suicidal ideation

This study also sought to better understand participant beliefs about aspects that support accurate SI reporting during EMA. Manifest content analysis (Kleinheksel et al., 2020), a form of qualitative content analysis, was conducted using qualitative free response data from the 3-month follow-up (i.e., “What, if anything, would have made you feel comfortable to report current suicidal thoughts on the daily surveys?”). Emergent coding was utilized in this study to identify observable categories within the text responses (Kleinheksel et al., 2020; Stemler, 2000). These observable categories were identified by reviewing the text responses and noting patterns in the data, while also accounting for unique perspectives that were expressed less frequently. Of the 80 participants, 43 (53.75 %) provided a codable response, 32 (40.00 %) did not provide a descriptive recommendation and were consequently not coded (e.g., “I’m not sure”, “N/A”, “I felt comfortable”), and 5 (6.25 %) did not respond.

Subsequently, one or more codes were assigned to each codable response to account for all aspects of provided feedback. Coded responses were then organized into categories, with one or more codes making up a category. See Table 5 for a list of categories. For example, a response of “If I could opt out of check-ins” was assigned a code corresponding to the category of ‘researcher check-in to evaluate suicide risk not required’. Likewise, responses of “I think maybe a little more information about the threshold that would lead to a contact” and “I think the researchers did a great job at priming me and reassuring me that there would be a check-in and conversation before suggesting (or requiring) hospitalization for suicidal thoughts” were each assigned different codes, both which corresponded to the category of ‘receiving detailed information about risk management (confidentiality, procedures, etc.)’. The number of responses falling within each category was counted and reported to summarize participant perspectives on approaches to support comfort with SI reporting during EMA research.

## 3. Results

### 3.1. EMA compliance and risk management

EMA compliance was 80.32 % for those who completed the post-EMA survey and 80.89 % for those who completed the 3-month survey. A total of 97.90 % of this sample (*n* = 92 unique individuals) reported any SI on an EMA survey, with 81 participants recalling this experience on the post-EMA survey. Across the entire sample, the independent decision to use crisis resources was reportedly made by four individuals (4.30 %) during the EMA period, with three citing a study-related influence on this decision (i.e., being a study participant, *n* = 2; receiving crisis resources from the app/study team, *n* = 2; completing a researcher check-in, *n* = 1).

The study team conducted 78 suicide risk check-ins with 33 unique participants during EMA data collection; 54.55 % (*n* = 18) of these participants created or reviewed a safety plan, and 0 % required a researcher facilitated police/mobile crisis unit involvement (to evaluate safety) or psychiatric hospitalization. To our knowledge, no participant was otherwise hospitalized during EMA. There were 21 participants who were contacted to complete a risk check-in but it was not completed due to the participant either returning contact (i.e., returned call/text) but choosing not to engage (*n* = 15) or due to the participant not replying to the contact (*n* = 6).

### 3.2. Aim 1: Evaluate concealing and underreporting suicidal ideation during EMA

Rates of contemplating and engaging in underreporting and concealing SI, as disclosed in the post-EMA survey, are reported in Table 2 for the overall sample and for those whose EMA reporting triggered a researcher check-in (i.e., active SI rated ≥3; rates reported by level of involvement with the research team – completed risk assessment, did not reply to contact, etc.). Among those who recalled experiencing any non-zero SI during an EMA survey (*n* = 81), 72.84 % (*n* = 59) reported having any thoughts of modified SI reporting (i.e., thoughts of underreporting *and/or* concealing) during the EMA period. Additionally, 59.30 % (*n* = 48) reportedly engaged in modified SI reporting (i.e., underreported *and/or* concealed SI) during EMA, with 32.10 % (*n* = 26) engaging in modified reporting repeatedly. Those who completed two or more check-ins had the highest rates of contemplating (76.50 %) and engaging in (64.70 %) SI underreporting, whereas those who expressed unavailability/disinterest in completing the check-in had the highest rates of contemplating (80.00 %) and engaging in (53.30 %) SI concealment. Those who met criteria for a risk check-in during the EMA period (i.e., rated SI intensity ≥3) reported higher rates of contemplating and engaging in underreporting and concealing SI than the overall sample of participants who experienced *any* SI during EMA reporting – with the exception of those who did not respond to researcher contact for a check-in the area of SI underreporting.

Please see Table 3 for a summary of endorsement rates from the post-EMA survey pertaining to motivations for having thoughts of concealing and underreporting SI during EMA; rates are reported for the overall sample and by level of researcher contact following an attempt to complete a risk check-in. Among the overall sample, not wanting researcher contact was the most commonly endorsed motivation for thinking about concealing or underreporting present SI during EMA completion (endorsed by 69.57 % to 79.59 %), followed by fear of a safety evaluation (i.e., “wellness check”) by emergency services (50.00 % to 55.10 %) and fear of involuntary hospitalization (endorsed by ~32.00 % for both categories). Free-text reporting indicated additional reasons for thoughts of concealing (TC) and thoughts of underreporting (TU), including experiencing negative emotions (e.g., burdensomeness, guilt, embarrassment; TC = 12.24 % [*n* = 6]; TU = 4.35 % [*n* = 2]), believing they were not a danger to themselves (TC = 6.12 % [*n* = 3]; TU = 6.52 % [*n* = 3]), and fear of study exclusion (TU = 2.17 % [*n* = 1]). When considering the subset that met criteria for a risk check-in during the EMA period, those who did not complete a check-in more frequently rated study team contact as a motivation to contemplate concealing SI (ranged from 91.70 % to 100 %), as compared to other groups (60.00 % to 83.30 %). Conversely, those who completed repeated check-ins less frequently endorsed that their thoughts of SI concealment were motivated by fear of involuntary hospitalization, as compared to other groups (25.00 % versus rates ranging from 33.30 % to 50.00 %).

Several correlates of thinking about and engaging in modified SI reporting during EMA were identified. Younger participants (large effect), those identifying as a sexual minority (small effect), and those more frequently reporting SI during EMA (medium effect) were more likely to think about modifying their SI responses. Additionally, younger (medium effect) and employed (small effect) participants and also those reporting a lower lifetime SI frequency (small-to-medium) were more likely to have engaged in modified SI reporting during EMA. All other results were non-significant. Bivariate model results are summarized in Table 1.

### 3.3. Aim 2: Investigate the influence of the EMA safety protocol on momentary suicidal thoughts and emotions

See Table 4 for results describing participants’ retrospective beliefs about the influence of completing a suicide risk check-in with a researcher during EMA. Approximately 68 % believed that, on average, completing a check-in decreased their suicidal thinking to some extent, with 20 % reporting a moderate decrease and 0 % reporting an extreme decrease. Additionally, 75 % reported experiencing decreases in negative thoughts and emotions to some degree, with 18 % reporting a moderate decrease and 6 % reporting an extreme decrease. Also, 63 % reported experiencing any increase in positive thoughts and emotions, with 15 % reporting a moderate increase and 9 % reporting an extreme increase. Participants generally estimated that these effects lasted minutes (reported by 26–30 %) to days (reported by 15–23 %) following the check-in, although changes in suicidal thinking were reported as longer-lasting (i.e., 40 % endorsed hours to days), whereas changes in positive thoughts and emotions were shorter-lived (i.e., 45 % reported effects were immediate or lasted ≤1 h).

Results from multi-level modeling indicated that the within-person SI by check-in interaction term was significant (*b* = −0.15 [95 % CI −0.22, − 0.06], *SE* = 0.04, *t* = −3.46). When an individual reported a higher-than-usual global SI score at *time t* and subsequently did not complete a check-in, on average, the SI score at the next time point (*t* + *1*) increased by 0.27 points more than when a check-in was completed (estimated marginal means [EMM], 5.22 [95 % CI = 4.96, 5.49] vs. 4.95 [95 % CI = 4.63, 5.28], respectively; *p* = .006), representing a small effect (*d* = 0.39). When within-person average (*p* = .30) or lower-than-usual SI scores (*p* = .89) were reported at *time t*, SI scores reported at *t* + *1* following a check-in, on average, did not significantly differ from scores reported when no check-in was completed.^1^

The within-person negative affect by check-in interaction term (*b* = −1.03 [95 % CI −0.28, 6.88], *SE* = 8.77, *p* = .24) and main effect of check-in (*b* = 6.62 [95 % CI = −0.08, 1.41], *SE* = 3.79, *p* = .08) were non-significant; however, there was a within-person positive association between negative affect at *time t* and *t* + *1* (*b* = 3.32 [95 % CI 0.29, 3.74], *SE* = 2.10, *p* < .001). The within-person positive affect by check-in interaction term was significant (*b* = −2.33 [95 % CI = −0.43, −0.04], *SE* = 9.93, *t* = −2.35, *p* = .02). When an individual reported a higher-than-usual positive affect score at *time t* and subsequently did not complete a check-in, on average, the affect score at the next time point (*t* + *1*) increased by 0.71 points more than when a check-in was completed (EMM, 4.63 [95 % CI = 4.33, 4.94] vs. 3.92 [95 % CI = 3.34, 4.50], respectively; *p* = .005), representing a small-to-medium effect (*d* = 0.49). When an individual reported an average positive affect score at *time t* and subsequently did not complete a check-in, on average the affect score at the next time point (*t* + *1*) increased by 0.48 points more than when a check-in was completed (EMM, 4.38 [95 % CI = 4.08, 4.68] vs. 3.90 [95 % CI = 3.42, 4.38], respectively; *p* = .01), representing a small effect (*d* = 0.33). When within-person lower-than-usual positive affect scores were reported at *time t*, positive affect scores reported at *t* + *1* following a check-in, on average, did not significantly differ from scores reported when no check-in was completed (*p* = .15).

### 3.4. Aim 3: Explore participant recommendations to increase comfort with reporting suicidal ideation

Table 5 provides a summary of participant recommendations to improve comfort with SI reporting during EMA, with 15 overarching categories of responses emerging based upon 43 participants. Text responses ranged in length from 6 to 103 words per participant, with an average length of 36 words and 79.07 % (*n* = 34) of participants providing a response ≥15 words. Among the most common types of responses, 23 (53.49 %) participants reported they would feel fully comfortable reporting SI during an EMA study only if certain safety procedures are not implemented or required (i.e., researcher check-in, safety evaluation (i.e., “wellness check”) by emergency service, psychiatric hospitalization). Some participants reported that they would be open to a modified safety protocol – such as the use of a streamlined risk check-in (9.30 %), a text message check-in (6.98 %), and no in-app ‘988’ resource reminders (2.33 %) – while others highlighted the importance of being given detailed information about the safety protocol (13.95 %). Conversely, 16.28 % provided a response indicating that receiving information about available mental health resources is an encouragement to accurately reporting SI during EMA and 6.98 % reported that knowing the research team would check in made them feel more comfortable about reporting SI. Participants also expressed that aspects of interacting with the research team increase comfort with reporting SI, including supportive and non-judgmental communication from the research staff (9.30 %) and receiving reassurance that they are not a burden to the study team (2.33 %).

## 4. Discussion

The current study extends our understanding of participant experiences during an EMA study employing a suicide risk safety protocol. First, this study investigated rates, rationale for, and correlates of contemplating and engaging in concealing and underreporting SI during EMA. Broadly, underreporting, concealing, and thoughts about this were quite common, certain groups reported higher rates, multiple concerns motivated these thoughts, and several characteristics influenced the likelihood of thinking about and engaging in underreporting and concealing. Second, investigating the impact of implemented safety procedures on participant SI and emotion states, this study found that completed suicide risk check-ins had small to medium effects on SI and positive affect under certain conditions, but not negative affect; although, many participants retrospectively believed the impact was more considerable and prolonged. Third, this study explored participant recommendations to increase comfort with SI reporting, with several categories of feedback emerging to inform the design of future EMA studies. These results highlight the challenges associated with studying real-time suicide risk, while providing promising future directions.

First, this study aimed to investigate rates, rationale for, and correlates of contemplating and engaging in concealing and underreporting SI during EMA – extending our understanding of participant experiences during an EMA study employing a suicide risk safety protocol. Approximately 75 % contemplated underreporting and/or concealing SI during EMA when SI was present and approximately 60 % concealed and/or underreported. These rates are notably higher than underreported suicidal intent (22 %; Bentley et al., 2024), although in line with studies of SI concealment outside of EMA (Blanchard and Farber, 2016; Love and Morgan, 2021). Participant discomfort in reporting SI is likely a common challenge facing EMA research, representing a complex participant concern and impediment to studying and monitoring real-time suicide risk. There was general participant agreement that researcher suicide risk check-ins were unwanted and this motivated thoughts about modifying SI responses; this study’s more conversative threshold for a check-in likely played a role in this result. Fear of a safety evaluation by emergency services was also a frequently cited motivation, with concerns of involuntary hospitalization also reported. Patients fear undesired outcomes (e.g., safety evaluation) even when reporting mild SI (Blanchard and Farber, 2016), which may partly explain these results. These findings are somewhat surprising given participants were provided with extensive protocol-related information to target anticipated barriers to reporting (e.g., address only temporarily recorded and deleted after each contact, EMA responses alone are insufficient to prompt safety evaluation by emergency services, confidentiality limits). Understanding and addressing participant concerns within future EMA research is imperative to improve participant comfort with SI reporting and to preserve ecological validity.

This study also provides preliminary insight into those most likely to think about and engage in modified SI reporting during EMA, including sexual minorities, younger and employed participants, those with less frequent SI history, and those more frequently reporting SI during EMA. Extant research offers possible explanations for these results. For example, individuals identifying as a sexual minority may have concerns about the nature of researcher check-ins and discrimination during possible intervention (Ayhan et al., 2020; Casey et al., 2019). Younger individuals may want to avoid a call due to anxiety (Bairwa et al., 2024; Turner, 2024), whereas employed participants may wish to avoid discussing private information while at work (Russinova et al., 2011). Greater discomfort discussing SI may also drive SI concealment for those with less frequent SI history (Mérelle et al., 2018). These identified participant groups would likely benefit from more extensive discussions surrounding their comfort with and barriers to SI reporting. Text-based check-ins may alleviate some concerns; this was only provided upon request in this study. Given suspected concerns about privacy, discomfort with, and the inconvenience of researcher contact, replacing researcher contact with in-app crisis resource reminders (e.g., Schatten et al., 2020) may improve rates of accurate SI reporting. Further research should explore possible functions of these findings and other potential correlates.

The second aim of this study was to evaluate changes in SI and emotion states following researcher-initiated suicide risk check-ins, representing the first study to evaluate the possible intervention effects of completed check-ins during a non-treatment EMA study. Completing a check-in had a small effect on SI when heightened SI was reported at the prior time point. Likewise, completing a check-in had small-to-medium effects on positive affect when heightened or typical levels of positive affect were previously reported. These findings suggest that check-ins may exert localized influence among those who participate in them, particularly during periods of heightened SI. However, these results suggest that risk check-ins may not produce clinically significant changes in SI or affect. For example, among participants reporting higher-than-usual SI prior to a check-in, SI ratings following a check-in were on average 0.27 points lower than when a check-in was not completed (e.g., 4.95 vs. 5.22, respectively). Notably, participants reported lower positive affect after a check-in than at comparable time points without a check-in. This may reflect a reduced opportunity to engage in mood-regulating activities, or it may indicate that participants did not experience the check-in as beneficial—and in some cases, may have felt worse afterward. Results should also be considered within the context of the conservative check-in threshold (i.e., ≥3 rating of active SI on the 5-point scale) used in this study. Researcher check-ins may have greater clinical utility during periods of higher suicide risk (e.g., severe SI intensity, suicidal plan and/or intent present); outside of this, a check-in may interrupt independent management of SI and affect and delay the natural decline of SI for some.

Interestingly, many participants retrospectively believed that completed contacts had positive, more substantial, and longer lasting impacts on SI, negative affect/thoughts, and positive affect/thoughts, although the extent and duration varied. Differences in EMA and post-EMA survey results may, at least in part, be an artifact of retrospective recall bias. Yet, it is also likely that the EMA data could not capture short-lived changes due to the survey spacing (3/day over 12 h), such as increased positive affect reported on the post-EMA survey. Overall, many participants believed that suicide risk assessment and safety planning were effective in mitigating real-time suicide risk and improving emotional well-being; yet, these procedures had relatively small lasting clinical effects as detected via EMA. Additional study, including replication, is needed to better understand the clinical meaningfulness of suicide risk check-ins more broadly.

Third, this study gathered participant recommendations for enhancing comfort with SI reporting during EMA. Participants provided rich insight into several strategies to mitigate underreporting and concealing SI during EMA. Many expressed a preference for safety protocols absent of researcher contact. Although, in-app crisis resource reminders (e.g., Schatten et al., 2020) – the alternative – are infrequently used by participants (Coppersmith et al., 2024), as in this study. One participant reported the in-app ‘988’ resource (i.e., National Suicide and Crisis Lifeline) reminder was “a deterrent for providing completely true data” due to “strong negative experiences”. Although not commonly reported, this emphasizes the value of conferring with individuals with lived experience when designing suicide research.

These findings have important implications for the development and ethical review of EMA safety protocols in suicide research. See Table 6 for a summary of researcher recommendations, informed by results from the current study, related to the design, conduct, and dissemination of future EMA research measuring SI. Participants frequently reported that certain standard procedures—such as phone call-based check-ins or the potential for emergency response—discouraged honest reporting of SI. This disconnection between protocol intent and participant experience underscores the need for Institutional Review Boards (IRBs) and researchers to support participant-centered approaches. Tailoring protocols to individual needs—such as offering check-in modality options (e.g., text vs. call), using clear consent scripts that demystify confidentiality and intervention thresholds, and consulting with individuals with lived experience during protocol development—may enhance participant comfort and disclosure. Importantly, our findings suggest that rigid or overly cautious safety procedures, while well-intentioned, may inadvertently reduce participant safety by suppressing accurate risk reporting. Likewise, study findings provide evidence that safety procedures within EMA research introduce some level of treatment effect, despite participants providing consent to engage in a non-treatment, observational study; this accentuates the ethical responsibility to provide participants with detailed, comprehensive information about the suicide risk management protocol during informed consent – including potential interventions the research team may implement. This finding also underlines the importance of designing EMA studies in a manner that balances participant safety with the intended goal of the study.

Moreover, the data highlight the need to consider demographic and developmental differences when designing risk management strategies. Younger participants, for instance, were more likely to underreport or conceal SI—likely reflecting anxiety about interpersonal contact, privacy concerns, or misunderstandings about protocol consequences. Rather than increasing protocol stringency for younger or higher-risk individuals, our results support developmentally sensitive approaches that prioritize transparency, flexibility, and participant autonomy. The recommendations put forth in this paper align with emerging clinical and research guidelines (e.g., Nock et al., 2021; Schatten et al., 2020; U. S. VA, 2024), which advocate for proportionate, collaborative responses to risk that distinguish between imminent crisis and chronic ideation. Integrating these ethical principles into EMA safety protocols may not only improve participant protection but also strengthen the scientific validity of data collected in suicide research.

Study limitations should be considered. This study had a relatively small sample (although large for EMA; Ammerman and Law, 2022), and as such heavily relied on summary statistics and simple models; results should be considered within this context and future research is needed to further shed light on the pervasiveness of the thoughts and behaviors studied herein. Likewise, future research utilizing qualitative interviews to understand participant perceptions about reporting SI during EMA studies may uncover a wider range and depth of information to inform researcher decisions surrounding study design. Bias due to social desirability and retrospective recall may have impacted the data. For example, some participants may not have chosen to report their past thoughts and behaviors of underreporting and concealing SI when completing the post-EMA survey. Likewise, being asked to appraise the influence of suicide risk check-ins following the EMA period, instead of during, may have led participants to recall a more pronounced impact than was previously experienced. Geographic context is also a consideration. Cultural norms and stigma surrounding the reporting of SI in the Midwestern U.S. (Alvarado-Torres et al., 2024; Dell et al., 2021; Diouf et al., 2022) may represent a source of social desirability bias – potentially influencing results. This study was unable to measure short-lived (e.g., lasting minutes) effects of a check-in given the EMA survey spacing (3/day over 12 h). Additionally, we were unable to study the impact of a check-in on the thoughts and emotions of those who qualified for a check-in but did not complete one. Finally, this sample had low racial diversity, yet a moderate to high degree of diversity in sexual orientation, gender, and age. Further replication is needed to better understand generalizability, given that inclusion criteria, sample size and characteristics, and safety protocols vary widely by EMA study (Ammerman and Law, 2022; Bentley et al., 2021).

## 5. Conclusion

The common occurrence of underreporting and concealing SI during EMA is an incredible challenge to protecting participants and collecting ecologically valid data. Contributions to these thoughts and behaviors appear multi-faceted, and likely deep-rooted. EMA safety protocols appear to have relatively small effects on participant SI and small-to-moderate effects on positive affect, although replication of findings is crucial. The recommendations for future research, informed by individuals with lived experience, offer suggestions to create a more comfortable environment for participants to report their authentic experiences. Many participants likely have prior negative experiences disclosing SI to mental health and health professionals (MacDonald et al., 2020; O’Keeffe et al., 2021). Consequently, suicidologists have the challenge and opportunity of building trust with participants, which may facilitate the real-time disclosure of suicidal thoughts and behaviors.

## Supplementary Material

Suicide Risk Management _ Supplemental Material

## Figures and Tables

**Table 1:** Sample characteristics associated with contemplating and engaging in modified reporting of suicidal ideation during EMA.**

Variable	Overall Sample (*N* = 94)	Thoughts of Modifying Suicidal Ideation Reporting (*N* = 81)				Modified Suicidal Ideation Reporting (*N* = 81)			
		Yes (*n* = 59)	No (*n* = 22)	Test Statistic (*df*)	Effect Size	Yes (*n* = 48)	No (*n* = 33)	Test Statistic (*df*)	Effect Size
Age, *M* (*SD*)	33.63 (11.87)	30.51 (9.52)	41.77 (14.60)	*t*(27.93) = −3.36	*d* = −1.02	30.58 (10.05)	37.91 (13.66)	*t*(79) = −2.78*	*d* = −0.62
Race (White), *n*(%)	74 (78.70 %)	47 (79.70 %)	18 (81.80 %)	^a^	V = 0.02	39 (81.30 %)	26 (78.80 %)	*χ*^2^(1) = 0.08	V = 0.03
Gender, *n*(%)				^a^	V = 0.15			*χ*^2^(2) = 0.67	V = 0.09
Female	59 (62.80 %)	34 (57.60 %)	16 (72.70 %)			29 (60.40 %)	21 (63.60 %)		
Male	20 (21.30 %)	14 (23.70 %)	4 (18.20 %)			10 (20.80 %)	8 (24.20 %)		
Non-binary/other	15 (16.00 %)	11 (18.60 %)	2 (9.10 %)			9 (18.80 %)	4 (12.10 %)		
Transgender (yes), *n*(%)	16 (17.00 %)	11 (18.60 %)	2 (9.10 %)	^a^	V = 0.12	9 (18.80 %)	4 (12.10 %)	*χ*^2^(1) = 0.64	V = 0.09
Sexual Orientation (heterosexual), *n*(%)	36 (38.30 %)	18 (30.50 %)	12 (54.50 %)	*χ*^2^(1) = 3.97*	V = 0.22	14 (29.20 %)	16 (48.50 %)	χ^2^(1) = 3.13	V = 0.20
Employment Status (employed), *n*(%)	65 (69.10 %)	43 (72.90 %)	12 (54.50 %)	*χ*^2^(1) = 2.47	V = 0.18	37 (77.10 %)	18 (54.50 %)	χ^2^(1) = 4.56*	V = 0.24
SI Age of Onset, *M* (*SD*)	13.64 (4.29)	14.05 (4.46)	13.09 (4.17)	*t*(79) = 0.88	*d* = 0.22	13.88 (4.50)	13.67 (4.26)	*t*(79) = 0.21	*d* = 0.05
SI Frequency History, *M* (*SD*)	1966.55 (2812.15)	1694 (2518)	2993 (3324)	*t*(79) = −1.89	*d* = −0.47	1514 (2377)	2821 (557)	*t*(79) = −2.11*	*d* = −0.48
Suicide Plan History (yes), *n*(%)	80 (85.10 %)	51 (86.40 %)	19 (86.40 %)	^a^	V = 0.001	42 (87.50 %)	28 (84.80 %)	^a^	V = 0.04
Suicide Attempt History (yes), *n*(%)	52 (55.30 %)	34 (57.60 %)	13 (59.10 %)	χ^2^(1) = 0.01	V = 0.01	28 (58.30 %)	19 (57.60 %)	*χ*^2^(1) = 0.005	V = 0.008
Percentage of EMA Prompts with SI endorsed, *M* (*SD*)	43.20 (30.59)	52.25 (29.54)	37.47 (26.95)	*t*(79) = 2.05*	*d* = 0.51	53.34 (29.18)	40.81 (28.65)	*t*(79) = 1.91	*d* = 0.43
Number of Suicide Risk Check-ins, *M* (*SD*)	0.83 (1.58)	1.08 (2.83)	0.59 (1.05)	*t*(79) = 1.19	*d* = 0.30	1.06 (1.78)	0.79 (1.50)	*t*(79) = 0.73	*d* = 0.16
EMA Response Rate, *M* (*SD*)	80.32 (14.98)	79.22 (13.09)	83.06 (18.10)	*t*(79) = −1.06	*d* = −0.26	79.63 (12.01)	81.17 (3.11)	*t*(51.52) = −0.43	*d* = −0.11
Outpatient Psychiatric Treatment– Past Year, *n*(%)	80 (85.10 %)	53 (89.80 %)	16 (72.70 %)	^a^	V = 0.21	42 (87.50 %)	27 (81.80 %)	^a^	V = 0.08
Number of Prior Psychiatric Hospitalizations, *M* (*SD*)	1.63 (3.31)	1.59 (3.11)	2.14 (4.36)	*t*(79) = −0.62	*d* = −0.15	1.42 (2.68)	2.21 (4.38)	*t*(79) = −1.01	*d* = −0.23

*Note. M*, mean; *SD*, standard deviation; *df*, degrees of freedom; *EMA*, ecological momentary assessment; *SI*, suicidal ideation;

a Fisher’s exact test does not calculate a test statistic

* *p* < .05.

** *p* < .001.

**Table 2:** Rates of contemplating and engaging in concealing and underreporting suicidal ideation during EMA.

Variable *n* (%)	Overall Sample^a^	Completed ≥2 check-ins (*n* = 17)	Completed 1 check-in (*n* = 15)^b^	Responded to researcher contact, check-in not completed^c^ (*n* = 15)	Did not respond to researcher contact, check-in not completed (*n* = 6)
Thoughts of concealing SI	49 (62.00 %)	12 (70.60 %)	10 (66.70 %)	12 (80.00 %)	4 (66.70 %)
Concealed SI	31 (39.20 %)	7 (41.20 %)	5 (33.30 %)	8 (53.30 %)	3 (50.00 %)
Thoughts of underreporting SI	46 (56.80 %)	13 (76.50 %)	9 (60.00 %)	11 (73.30 %)	3 (50.00 %)
Underreported SI	37 (45.70 %)	11 (64.70 %)	7 (46.70 %)	9 (60.00 %)	2 (33.33 %)

*Note*. SI, suicidal ideation; *EMA*, ecological momentary assessment; *n* = total number of cases in a group; SRA, suicide risk assessment.

a Endorsement rates from those who completed the post-EMA survey and recalled experiencing any non-zero SI during an EMA survey, *N* = 79 for concealment thoughts/behaviors, *N* = 81 for underreporting thoughts/behaviors.

b One additional participant had missing data

c These individuals told the researcher they were unavailable and/or disinterested in completing a check-in; Concealing, defined as denying suicidal ideation despite its presence; Underreporting, defined as under-rating the intensity of present suicidal ideation.

**Table 3:** Cited reasons for having thoughts of concealing and underreporting suicidal ideation during EMA.

Variable *n* (%)	Overall Sample^a^		Completed ≥2 check-ins (*n* = 17)		Completed 1 check-ins (*n* = 15)^b^		Responded to researcher contact, check-in not completed^c^ (*n* = 15)		Did not respond to researcher contact, check-in not completed (*n* = 6)	
	Thoughts of Concealing SI (*N* = 49)	Thoughts of Underreporting SI (*N* = 46)	Thoughts of Concealing SI (*n* = 12)	Thoughts of Underreporting SI (*n* = 13)	Thoughts of Concealing SI (*n* = 10)	Thoughts of Underreporting SI (*n* = 9)	Thoughts of Concealing SI (*n* = 12)	Thoughts of Underreporting SI (*n* = 11)	Thoughts of Concealing SI (*n* = 4)	Thoughts of Underreporting SI (*n* = 3)
Did not want to receive a check-in contact from the study team	39 (79.59 %)	32 (69.57 %)	10 (83.30 %)	11 (84.60 %)	6 (60.00 %)	4 (44.40 %)	11 (91.70 %)	8 (72.70 %)	4 (100.00 %)	3 (100.00 %)
Afraid of safety evaluation (e.g., “wellness check”) by emergency services	27 (55.10 %)	23 (50.00 %)	5 (41.70 %)	5 38.50 %)	6 (60.00 %)	6 (66.70 %)	5 (41.70 %)	5 (45.50 %)	3 (75.00 %)	1 (33.30 %)
Afraid of involuntary hospitalization	16 (32.65 %)	15 (32.61 %)	3 (25.00 %)	3 (23.10 %)	4 (40.00 %)	5 (55.60 %)	4 (33.30 %)	3 (27.30 %)	2 (50.00 %)	1 (33.30 %)

*Note*. SI, suicidal ideation; *EMA*, ecological momentary assessment; *n* = total number of group cases.

a Endorsement rates from those who completed the post-EMA survey and recalled experiencing any non-zero SI during an EMA survey, *N* = 79 for concealment thoughts/behaviors, *N* = 81 for underreporting thoughts/behaviors.

b One additional participant had missing data.

c These individuals told the researcher they were unavailable and/or disinterested in completing a check-in; participants could endorse multiple reasons for their thoughts; Concealing, defined as denying suicidal ideation despite its presence; Underreporting, defined as under-rating the intensity of present suicidal ideation.

**Table 4:** Participant retrospective beliefs about the influence of completing a suicide risk check-in.

	Decreased Suicidal Thinking (*N* = 30)^a^	Decreased Negative Thoughts and Emotions (*N* = 33)	Increased Positive Thoughts and Emotions (*N* = 33)
Extent of Influence			
Not at all	10 (33.33 %)	8 (24.20 %)	12 (36.40 %)
Slightly	7 (23.33 %)	11 (33.30 %)	9 (27.30 %)
Somewhat	7 (23.33 %)	6 (18.20 %	4 (12.10 %)
Moderately	6 (20.00 %)	6 (18.20 %)	5 (15.20 %)
Extremely	0	2 (6.10 %)	3 (9.10 %)
Duration of Influence			
Immediate Influence^b^	0	4 (12.10 %)	5 (15.10 %)
2–15 min afterwards	3 (10.00 %)	6 (18.20 %)	3 (9.10 %)
16–60 min afterwards	5 (16.70 %)	3 (9.10 %)	7 (21.20 %)
Less than 1 day afterwards	5 (16.70 %)	7 (21.20 %)	1 (3.00 %)
1 day or more afterwards	7 (23.30 %)	5 (15.20 %)	5 (15.10 %)

a Three additional participants reported they were no longer experiencing suicidal thoughts at the time of the researcher check-in.

b During and within a minute after the check-in.

**Table 5:** Participant recommendations to increase comfort with reporting suicidal ideation during EMA.

Recommendation Category	*n* (%)
Researcher check-in to evaluate suicide risk not required	13 (30.23 %)
Threat of emergency service visit (e.g., “wellness check”) reduced or removed	8 (18.60 %)
Knowing there are resources available to help	7 (16.28 %)
Receiving detailed information about the safety protocol	6 (13.95 %)
Receiving information about what would happen if the participant did not answer the researcher check-in contact	1
Receiving information about what triggers a researcher check-in	1
Receiving detailed information about what to expect during a researcher check-in	2
Receiving detailed information about confidentiality as it relates to safety procedures	5
Threat of psychiatric hospitalization reduced or removed	4 (9.30 %)
Streamlined check-in with researcher instead of full suicide risk assessment	4 (9.30 %)
Supportive and non-judgmental research staff	4 (9.30 %)
Recommendation related to the EMA assessment of suicidal ideation	4 (9.30 %)
Assess if suicidal ideation is intrusive	1
Assess intention to act on suicidal ideation	1
Free response box to further describe suicidal ideation	1
Multiple items to evaluate passive suicidal ideation	1
Knowing the research team will check in	3 (6.98 %)
Option of receiving a text message check-in instead of a call	3 (6.98 %)
Knowing that I am sharing my experience to help others	2 (4.65 %)
Reassurance the participant is not a burden on the study team	1 (2.33 %)
Reassurance that high ratings of suicidal ideation will not result in participant exclusion	1 (2.33 %)
No in-app 988 resource reminder	1 (2.33 %)
The survey can be discretely completed around others	1 (2.33 %)

*Note*. EMA, ecological momentary assessment; the percentage was calculated based upon the number of participants (*n* = 43) providing a recommendation; participants could report multiple types of recommendations.

**Table 6:** Recommendations for future suicide research utilizing ecological momentary assessment.

Using automated crisis resources and messages directing participants to seek help (for full details, Schatten et al., 2020) is one possible approach to balancing participant preferences, participant safety, and ecological validity. When monitoring and responding to real-time risk, use imminent suicide risk markers as impetus for participant contact to minimize researcher intervention during non-crisis states (e.g., recent/current suicide plan, for a review see Bentley et al., 2021; intent rating ≥ 8/10, for complete procedures please see Bentley et al., 2024; for a multi-item approach, see Glenn et al., 2020). Provide the option to complete suicide risk check-ins by telephone call or text. Use a script to provide consistent, comprehensive information pertaining to confidentiality limits, clinical expertise, and the suicide risk management protocol (e.g., check-in format, content, and duration; steps taken if unsuccessful contact; collection/use of address data) and provide a written summary initially and mid-way through participation (e.g., when sending updates about EMA response rate). Reserve ample time during the informed consent process to (a) reassure participants that individuals who frequently have suicidal thoughts and meet check-in criteria are not a burden to staff and (b) directly ask about concerns regarding accurately reporting suicidal ideation, discuss and answer questions regarding personal barriers to reporting. Provide the option to receive a summary of study findings – tangible evidence of participant research contributions. Use a follow-up survey to evaluate suicidal ideation underreporting and concealing.

## Data Availability

Data available on request from the authors.

## Footnotes

1 When a sum score of the two active SI items was used in replacement of the global SI score as a predictor and outcome, the within-person SI by check-in interaction remained significant (*b* = −0.09 [95 % CI −0.15, −0.03], *SE* = 0.03, *t* = −3.08), with lower SI scores reported after a check-in, versus when one was not completed, following the reporting of higher-than-usual (estimated marginal means [EMM], 2.34 [95 % CI = 2.18, 2.51] vs. 2.66 [95 % CI = 2.51, 2.80], *p* < .001) and average active SI scores (EMM, 2.17 [95 % CI = 2.03, 2.31] vs. 2.39 [95 % CI = 2.29, 2.50], *p* < .001) at time *t*.

## Appendix A. Supplementary data

Supplementary data to this article can be found online at https://doi.org/10.1016/j.jad.2025.120361.
