# Supplementary material for: Suicide Risk Management in Ecological Momentary Assessment: Participant Concealment of Suicidal Thoughts and Experiences with Safety Procedures
Source: J Affect Disord. Author manuscript; Available in PMC 2025 Oct 17. (PMC12530944; doi:10.1016/j.jad.2025.120361)
Supplement: Suicide Risk Management _ Supplemental Material [file NIHMS2117373-supplement-Suicide_Risk_Management___Supplemental_Material.pdf]

Supplemental Material 1

Script Utilized during Informed Consent to Review Confidentiality Limits, the Suicide Risk Management Protocol, and Research Team's Clinical Expertise

> Third, and probably most relevant to this study, if someone reports serious thoughts, plan, and intention to act on thoughts of killing themselves in the very short-term, we would need to intervene to keep the person safe. We understand that people can experience thoughts of killing themselves even all throughout the day and it doesn't mean that that someone is automatically meeting this really high threshold set by the law. I like to sit on this point for a moment because we include questions that ask about current suicidal thoughts on the daily surveys. As part of our ethical responsibility for doing this research, we regularly monitor the data and will reach out by phone call if we see that someone has reported suicidal thoughts at a certain intensity. If we reach out by phone, it does not mean that we are automatically assuming that the high threshold set by the law is being met, and we are not automatically assuming we will have to break confidentiality. Rather, it is just a threshold we have set in our lab to just check in on the person, ask some questions to see how the person is doing, and offer support and resources. Most of the time this is all the call entails and most of the time these calls are brief. It has been rare that the high threshold set by the law has been met and that we have needed to take further steps to keep the person safe, such as talk to the person about willingness to go to the hospital. I also like to note that there are four of us in the lab who complete these check-in calls and that all of us are trained therapists and also study suicide, so we all feel very comfortable talking about suicidal thoughts and are not overly reactive, in my opinion, to hearing someone report that they are having serious suicidal thoughts, even with a plan and intent to act. If we can keep someone safe *and* keep them out of the hospital, this will always be our goal. Do you have any questions or concerns about this information?

The decision to add greater detail to this informed consent script was made approximately 2 weeks into study enrollment, given the perceived benefit to participants and given that participants were commonly asking what would happen if they did not answer the researcher's contact (e.g., phone call). Thus, the majority of participants were also told:

> As mentioned, the law indicates that we are required to intervene if someone reports serious thoughts, plan, and intention to act. Our daily surveys only ask about suicidal thoughts, not about whether someone has a suicide plan or intention to act. Due to this, if we are unable to reach someone by phone call, we will provide mental health resources but we would not have enough information to act further.

Supplemental Material 2
Participant Survey Following Ecological Momentary Assessment Completion – Evaluation of Experiences with Suicide Risk Safety Procedures and Reporting Suicidal Ideation

Thank you for your willingness to complete this survey! Your responses are important to us and will help us better understand your experience as a participant in the ELE study.

Please enter your study ID: ______

1. Did you use mental health crisis resources (i.e., suicide prevention lifeline or text line [988], 911, emergency department, etc.) during the 30-day period of smartphone surveys?
   Yes
   No

   BRANCH to 1a if answered 'yes' to question 1

   1a. Did *any* of the following influence your decision in ***some*** way to use mental health crisis resources during study participation? **Select all that apply.**
   Being a study participant.
   Receiving crisis resources from the smartphone app or study team.
   Completing a check-in contact with a member of the study team.
   Creating a safety plan with the study team.
   Reviewing an existing safety plan with the study team.
   None of the above.

2. Were you **ever hesitant** to report suicidal thoughts on the smartphone surveys during the 30-day period? ***Please carefully read these answer options.***
   I experienced suicidal thoughts at the moment when completing **any** of the smartphone surveys over the 30 day period **and** I **was hesitant at some point** to report my suicidal thoughts.
   I experienced suicidal thoughts at the moment when completing **any** of the smartphone surveys over the 30 day period **and** I **was not hesitant** at some point to report my suicidal thoughts.
   I did **not** experience suicidal thoughts at the moment when completing **any** of the smartphone surveys over the 30 day period **but** if I had experienced suicidal thoughts I imagine I **would have been hesitant** to report them
   I did **not** experience suicidal thoughts at the moment when completing **any** of the smartphone surveys over the 30 day period **but** if I had experienced suicidal thoughts I imagine I **would not have been hesitant** to report them

   BRANCH to 2a if answered question 2 with option #1 (experienced suicidal thoughts, was hesitant)

2a. Considering your hesitancy to report suicidal thoughts during the 30-day period of smartphone surveys…
On only one occasion I experienced suicidal thoughts at the time I was completing a smartphone survey but did not report it.
More than once I experienced suicidal thoughts at the time I was completing a smartphone survey but did not report it.
I always reported suicidal thoughts on the smartphone surveys when I had them.

BRANCH to 2ai if selected option #1 or #2 (once, more than once) for question 2a

2ai. **At any point during the study** did ***any*** of the following contribute to your hesitation **to report** suicidal thoughts during the 30-day period of smartphone surveys?
Select all that apply.

I did not want to receive a check in contact from the study team.
I was afraid that reporting my suicidal thought(s) would result in the police/emergency responders coming to my home to check on me.
I was afraid my that reporting my suicidal thought(s) would result in being hospitalized against my will.
Some other reason: ____________ (please describe)

3. Did you **ever consider underreporting** your suicidal thoughts on the smartphone surveys during the 30-day period (for example, considered reporting a lower number to describe how intense your suicidal thoughts were at the time than was accurate to your experience)? ***Please carefully read these answer options.***
I experienced suicidal thoughts at the moment when completing **any** of the smartphone surveys over the 30 day period **and** I **considered underreporting** my suicidal thoughts **at some point**.
I experienced suicidal thoughts at the moment when completing **any** of the smartphone surveys over the 30 day period **and** I **did not consider underreporting** my suicidal thoughts **at some point.**
I did **not** experience suicidal thoughts at the moment when completing **any** of the smartphone surveys over the 30 day period **but** if I had experienced suicidal thoughts I imagine I **would have** considered underreporting.
I did **not** experience suicidal thoughts at the moment when completing **any** of the smartphone surveys over the 30 day period **but** if I had experienced suicidal thoughts I imagine I **would not have considered** underreporting

BRANCH to 3a if selected option #1 (experienced suicidal thoughts, considered underreporting) for question 3

3a. Considering your thought(s) of underreporting suicidal thoughts during the 30-day period of smartphone surveys…
On only one occasion I rated my suicidal thoughts as less intense on the daily surveys than it actually was at the time.

<u>More than once</u> I rated my suicidal thoughts as less intense on the daily surveys than it actually was at the time.
I <u>always</u> reported the exact intensity of my suicidal thoughts on the daily surveys.
I did not experience suicidal thoughts at the moment of completing any of the smartphone surveys over the 30 day period.

Branch to 3ai if selected option #1 or #2 (once, more than once) to 3a

3ai. **At any point during the study** did ***any*** of the following contribute to your desire **to underreport** suicidal thoughts during the 30-day period of smartphone surveys?
<u>Select all that apply.</u>
I did not want to receive a check-in contact from the study team.
I was afraid that reporting my suicidal thought(s) would result in the police/emergency responders coming to my home to check on me.
I was afraid my that reporting my suicidal thought(s) would result in being hospitalized against my will.
Some other reason: ______________ (please describe below)

4. During the 30-day period of smartphone surveys, did a member of the study team contact you ***to check in about recent suicidal thoughts***?
   Yes, the study team **attempted** to contact me, but I did **<u>not ever</u>** complete a check-in.
   Yes, I was **<u>contacted and completed a check-in</u>** with a member of the study team **at least once.**
   No, a member of the study team **<u>did not</u>** attempt to contact me (outside of information about study logistics – for example, how many surveys I had completed).

If answer to #4 was 'Yes, contacted and completed a check-in' – BRANCH to questions #5-9
If answer to #4 was 'Yes, attempted contact, did not ever complete check-in' – BRANCH to questions #10-12

5. Did the study team member help you create a safety plan during your check-in?
   Yes/No

6. During a check-in with the study team, did you review a safety plan you had made in the past?
   Yes/No

7. On average, completing a check-in about suicidal thoughts with a member of the study team *decreased* my suicidal thinking.
   Not at all
   Slightly
   Somewhat
   Moderately
   Extremely

There was never a time that I was experiencing suicidal thoughts at the moment when I received a check-in.

BRANCH to 7a if selected 'Slightly' to 'Extremely' to question 7

7a. On average, the decrease in my suicidal thoughts lasted…
Only during the check-in with the study team member
1-60 seconds after the check-in
2-15 minutes after the check-in
16-60 minutes after the check-in
Less than one day after the check-in
1-2 days after the check-in
More than 2 days after the check-in

8. On average, completing a check-in about suicidal thoughts with a member of the study team *decreased* my negative thoughts or emotions.
Not at all
Slightly
Somewhat
Moderately
Extremely

BRANCH to 8a if selected 'Slightly' to 'Extremely' to question 8

8a. On average, the decrease in my negative thoughts or emotions lasted…
Only during the check-in with the study team member
1-60 seconds after the check-in
2-15 minutes after the check-in
16-60 minutes after the check-in
Less than one day after the check-in
1-2 days after the check-in
More than 2 days after the check-in

9. On average, completing a check-in about suicidal thoughts with a member of the study team *increased* my positive thoughts or emotions.
Not at all
Slightly
Somewhat
Moderately
Extremely

BRANCH to 9a if selected 'Slightly' to 'Extremely' to question 9

9a. On average, the increase in my positive thoughts or emotions lasted…
Only during the check-in with the study team member
1-60 seconds after the check-in

2-15 minutes after the check-in
16-60 minutes after the check-in
Less than one day after the check-in
1-2 days after the check-in
More than 2 days after the check-in

10. On average, the study team's ***attempt(s)*** to check in with me *decreased* my suicidal thinking.
Not at all
Slightly
Somewhat
Moderately
Extremely
There was never a time that I was experiencing suicidal thoughts at the moment when the study team attempted to contact me.

BRANCH to 10a if selected 'Slightly' to 'Extremely' to question 10

10a. On average, the decrease in my suicidal thoughts lasted…
1-60 seconds after the study team *attempted* to contact me.
2-15 minutes after the study team *attempted* to contact me.
16-60 minutes after the study team *attempted* to contact me.
Less than one day after the study team *attempted* to contact me.
1-2 days after the study team *attempted* to contact me.
More than 2 days after the study team *attempted* to contact me.

11. On average, the study team's ***attempt(s)*** to check in with me *decreased* my negative thoughts or emotions.
Not at all
Slightly
Somewhat
Moderately
Extremely

BRANCH to 11a if selected 'Slightly' to 'Extremely' to question 11

11a. On average, the decrease in my negative thoughts or emotions lasted…
1-60 seconds after the study team *attempted* to contact me.
2-15 minutes after the study team *attempted* to contact me.
16-60 minutes after the study team *attempted* to contact me.
Less than one day after the study team *attempted* to contact me.
1-2 days after the study team *attempted* to contact me.
More than 2 days after the study team *attempted* to contact me.

12. On average, the study team's ***attempt(s)*** to check in with me *increased* my positive thoughts or emotions.
Not at all
Slightly
Somewhat
Moderately
Extremely

BRANCH to 12a if selected 'Slightly' to 'Extremely' to question 12

12a. On average, the increase in my positive thoughts or emotions lasted…
1-60 seconds after the study team *attempted* to contact me.
2-15 minutes after the study team *attempted* to contact me.
16-60 minutes after the study team *attempted* to contact me.
Less than one day after the study team *attempted* to contact me.
1-2 days after the study team *attempted* to contact me.
More than 2 days after the study team *attempted* to contact me.
